# Fabricating a Cu-MBG-incorporated polyurethane foam with antibacterial properties and bioactivity for diabetic wound healing

**DOI:** 10.1016/j.isci.2026.116423

**Published:** 2026-06-17

**Authors:** Linqin Xie, Deng Huang, Kai Chen, Bangxuan Zhu, Yangkai Zhao, Jieke Li, Shouyi Wang, Mingliang Wu, Yili Xiang

**Affiliations:** 1 The Department of Interventional Vascular Surgery, The Third Clinical College of Wenzhou Medical University, Wenzhou People’s Hospital, Wenzhou, Zhejiang, China; 2 The Department of Infectious Disease, The Third Clinical College of Wenzhou Medical University, Wenzhou People’s Hospital, Wenzhou, Zhejiang, China; 3 The Department of Cardiovascular and Thoracic Surgery, The Second Affiliated Hospital & Yuying Children's Hospital of Wenzhou Medical University, Wenzhou, Zhejiang, China

**Keywords:** bioengineering, biomedical engineering, biomaterials, materials application

## Abstract

Chronic diabetic wounds require dressings that combine high liquid absorption, antibacterial activity, and bioactivity to support tissue repair. Here, copper-containing mesoporous bioactive glass (Cu-MBG) was incorporated into polyurethane foam to create a bioactive composite dressing (PU-Cu-MBG). The resulting foam exhibited strong liquid absorption, suitable water vapor transmission, and robust mechanical properties. The Cu-MBG endowed the dressing with antibacterial activity (>99% against *Staphylococcus aureus* and *Escherichia coli*) and promoted the proliferation of human endothelial cells. *In vivo*, the PU-Cu-MBG dressing enhanced angiogenesis, collagen deposition, and accelerated wound closure compared with commercial dressings in a diabetic skin wound model. These findings demonstrate that bioactive glass-loaded foam dressings can effectively combine physical and biological functions, offering a promising approach for the management of chronic diabetic wounds.

## Highlights

• Cu-MBG nanoparticles were uniformly incorporated into polyurethane foam
• PU-Cu-MBG foam showed high absorption, suitable WVTR, and robust mechanics
• PU-Cu-MBG achieved >99% antibacterial activity against *S. aureus* and *E. coli*
• PU-Cu-MBG accelerated diabetic wound healing via angiogenesis and collagen deposition

## Graphical abstract

**Figure:**
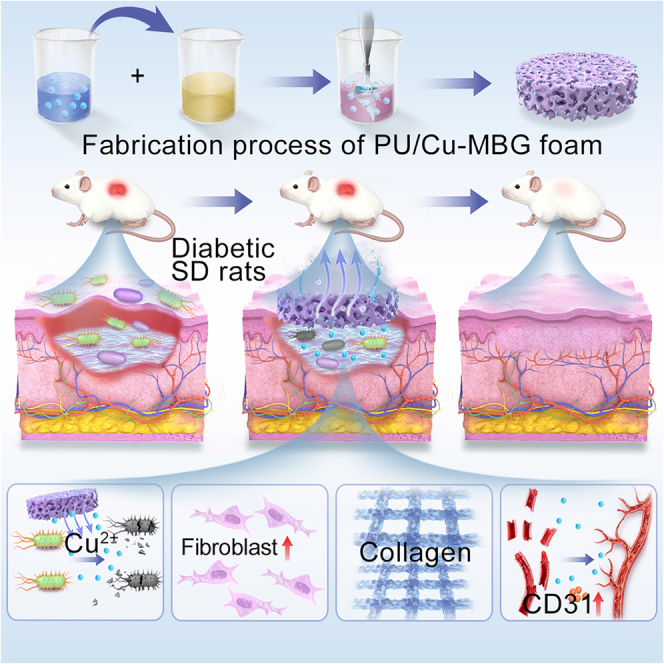

## Introduction

Chronic wounds caused by burns, pressure ulcers, immune deficiency, and diabetes often result in pain and infection.^1^^,^^2^ Their pathogenesis is complex and treatment duration is typically prolonged (exceeding one month), with associated costs being high, which causes significant distress to patients. The general process of wound healing includes the following stages: (1) hemostasis phase, (2) blood clotting phase, (3) inflammatory phase, (4) granulation tissue formation phase, (5) epithelialization phase, and (6) maturation phase.^3^^,^^4^^,^^5^ Treatment for chronic wounds typically involves a combination of surgery, medication, and wound dressings.^6^ Among these, wound dressings serve as temporary substitutes for damaged skin, helping to isolate bacteria, protect the wound, and provide a temporary matrix for cell migration, extracellular matrix deposition, and neovascularization.^7^^,^^8^^,^^9^

In the past few decades, numerous materials have become available for the treatment of chronic wounds in the market, for example, gauze, hydrocolloids, hydrogels, foam dressings, alginate dressings, and so on.^10^^,^^11^^,^^12^^,^^13^ Among them, hydrogel dressings have been widely developed due to their ability to provide a moist environment at the wound site, promoting tissue regeneration through granulation and re-epithelialization.^14^ Furthermore, their flexible and tunable properties allow for the incorporation of cells, antibacterial agents, growth factors, and biomolecules to accelerate wound contraction and healing.^15^ However, conventional hydrogels exhibit poor fluid absorption, while chronic wounds often produce excessive exudate that can cause leakage and contamination, thereby increasing the risk of infection and inflammation.^16^ In comparison, polyurethane (PU) foam with a porous structure demonstrates excellent absorbency for tissue exudate, robust mechanical properties, and good biocompatibility, making it widely used in chronic wound care and treatment.^17^^,^^18^ For example, Chen et al. prepared a high-absorption PU foam dressing modified by polyethylene glycol and triethoxysilane to promote wound healing through micro-negative pressure generated by its high absorption capacity.^19^ However, relying solely on the physical effects of foam dressings is insufficient, as chronic wounds remain at high risk of microbial infection, particularly by *Staphylococcus aureus*. Once bacteria attach to the wound tissue, they rapidly colonize and spread to organs, leading to severe complications such as septicemia and amputation.^20^ Furthermore, impaired angiogenesis is another critical issue in chronic wound healing. Without adequate vascularization, cells and tissues lack oxygen and nutrients, leading to delayed repair.^21^ Due to their 3D porous network, foams can be loaded with antimicrobial and bioactive agents, which are released into wound tissues to kill bacteria and accelerate neovascularization.^22^^,^^23^ However, the conventional antimicrobial agents such as antibiotics can cause the emergence of drug-resistance bacteria, while silver ions commonly exhibit some cytotoxicity. Additionally, approaches to improve bioactivity, including the use of growth factors or cell co-culture, are often limited by challenges related to production costs, quality control, and storage stability.

Recent research has found that inorganic materials composed of human nutritional elements possess innate bioactivity, and silicon (Si)-based biomaterials represent such a category of biomaterials.^24^^,^^25^ Mesoporous bioactive glass (MBG), for instance, has been shown to stimulate angiogenesis by continuously releasing silicon (Si) ions, which activate various tissue cells and regulate immune cells.^26^ As a result, they were used either on its own or in combination with polymer materials through various methods to produce fibers, films, and hydrogels for tissue regeneration.^27^^,^^28^^,^^29^^,^^30^ Moreover, MBG can act as a platform for integrating various bio-active metal ions (e.g., zinc, copper, cobalt, etc.) in the synthesis process to achieve a series of beneficial biological reactions through ionic dissolution products.^31^ For example, copper-containing MBG (Cu-MBG) possesses broad-spectrum antibacterial properties due to its ability to release copper ions (Cu^2+^). Cu^2+^ are also an essential mediator of angiogenesis, interacting with multiple factors in the wound healing cascade to promote repair.^32^^,^^33^ Meanwhile, the synergy between Si ions and Cu^2+^ can generate a stronger angiogenic effect, accelerating the repair of chronic wounds.^34^ Although Cu-MBG-loaded hydrogels have been recently reported for regenerative medicine applications, their relatively weak mechanical properties and limited fluid handling capacity may restrict their practical use in chronic wound management.^35^^,^^36^ Inspired by the above research, we speculate that the Si-based nanoparticles (NPs) could be encapsulated into foam dressings, and the combined physical action of the dressing and the biological efficacy of NPs may synergistically enhance the regulation of chronic wound repair. To the best of our knowledge, development of a Cu-MBG-incorporated PU foam for the treatment of diabetic wound has not been investigated.

**Scheme 1:**
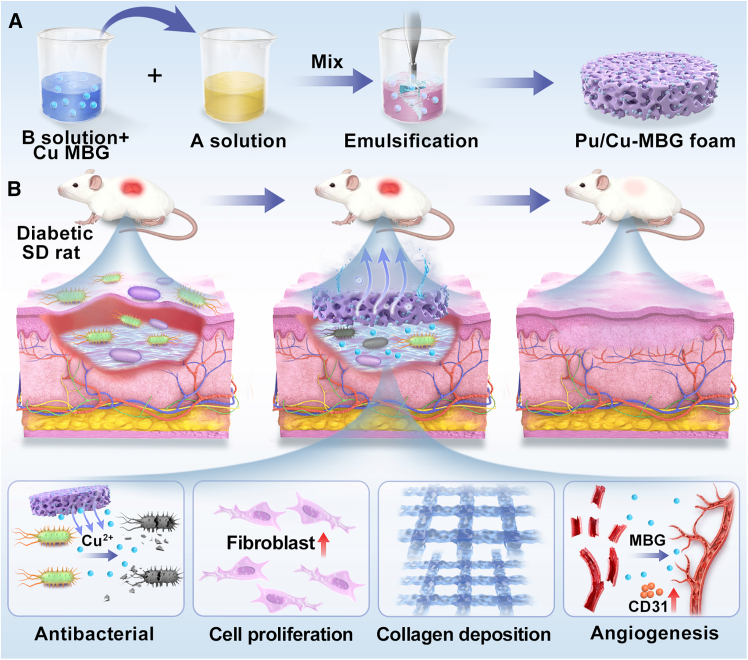
Schematic of the fabrication and application of PU-Cu-MBG foam (A) Fabrication process of PU-Cu-MBG foam. (B) Application of PU-Cu-MBG foam in a diabetic rat skin defect model.

Hence, a microemulsion-sol-gel method was employed to prepare Cu-MBG NPs, which were subsequently incorporated into hydrophilic PU foam dressings via emulsification and foaming (Scheme 1). The resulting composite foam was systematically characterized for its microstructure, mechanical properties, and physicochemical performance. Its antibacterial activity was evaluated against *Escherichia coli* (Gram-negative) and *S. aureus* (Gram-positive). Finally, the wound healing efficacy of the PU-Cu-MBG foam was investigated in a full-thickness skin defect model of diabetic Sprague Dawley (SD) rats. As illustrated in Scheme 1, the PU-Cu-MBG foam dressing combines the physical advantages of the porous foam matrix—including high liquid absorption capacity and moderate water vapor permeability—with the biological functions of the released Cu^2+^, which provide antibacterial protection against wound pathogens and promote tissue regeneration by stimulating fibroblast proliferation, collagen deposition, and angiogenesis. With its integrated physicochemical properties and bioactive functions, this composite foam represents a promising multifunctional dressing strategy for the management of chronic wounds, particularly diabetic ulcers.

## Results

### Synthesis and characterization of Cu-MBG NPs

**Figure 1:**
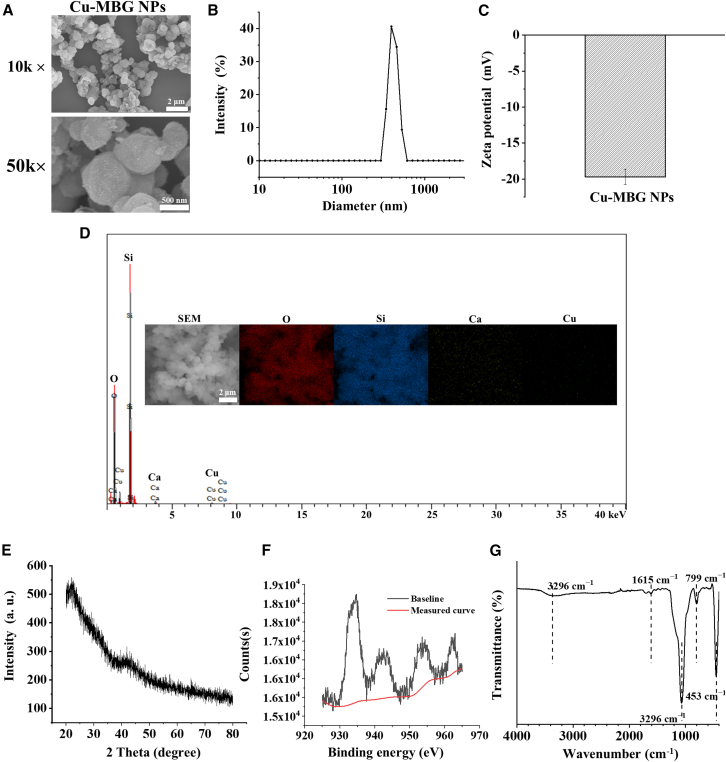
Characterization of Cu-MBG NPs (A) SEM images of Cu-MBG in 10k × (scale bars: 2 μm) and 50k × (scale bars: 500 nm) magnifications. (B) The particle size distribution of Cu-MBG. (C) The zeta potential of Cu-MBG. (D) The EDS spectra of Cu-MBG (scale bars: 2 μm). (E) The XRD pattern of Cu-MBG. (F) The Cu 2p spectra of Cu-MBG. (G) The FTIR spectra of Cu-MBG.

Cu-MBG NPs were obtained using the sol-gel method. Scanning electron microscopy (SEM) was employed to evaluate the morphology of the Cu-MBG NPs, revealing that all samples consisted of spherical particles with smooth surfaces and relatively uniform sizes ranging from 200 to 500 nm (Figure 1A). Besides, the average size of Cu-MBG NPs was determined by dynamic light scattering (DLS) with 439.14 ± 60.87 nm (Figure 1B). The zeta potential of the NPs was also measured. As depicted in Figure 1C, the surface zeta potential of Cu-MBG NPs was −19.70 ± 1.08 mV. Subsequently, energy-dispersive X-ray spectroscopy (EDS) analysis (Figure 1D) was conducted to examine the elemental composition, which confirmed the presence of O, Si, Ca, and Cu in the NPs, with the total Cu^2+^ content being 4.50 (wt %).

The crystalline structure of the NPs was further investigated using X-ray diffraction (XRD). As illustrated in Figure 1E, the diffraction peaks were broad and lacked sharp characteristic peaks, indicating that the bio-glasses obtained after high-temperature sintering were amorphous inorganic materials. In addition, the incorporated Cu^2+^ also exist in the free ion form within the structure of the bio-glass.^37^

Next, the X-ray photoelectron spectroscopy (XPS) analysis was performed on the Cu-MBG NPs. As depicted in Figure S1, the full survey spectrum confirmed the presence of Cu, along with the expected matrix elements. The high-resolution Cu 2p spectrum provided definitive evidence for the successful doping of copper (Figure 1F). Specifically, two distinct characteristic peaks were observed at binding energies of 933 and 953 eV, corresponding to Cu 2p_3_/_2_ and Cu 2p_1_/_2_, respectively. Furthermore, the presence of prominent shake-up satellite peaks at approximately 942 and 962 eV is a hallmark feature of the Cu^2+^ oxidation state. These spectroscopic features collectively and conclusively demonstrate that copper was successfully incorporated into the MBG matrix and existed primarily in a bivalent form (Cu^2+^).^38^

Fourier transform infrared (FTIR) spectroscopy was utilized to determine the structural properties of the Cu-MBG NPs within the range of 400–4000 cm^−1^. As shown in Figure 1G, a prominent absorption peaks at 3,350 and 1,615 cm^−1^ correspond to the –OH groups adsorbed on the sample surface. Most absorption bands around 453 and 799 cm^−1^ are attributed to the Si–O bending vibration and the symmetric stretching vibration of Si–*O*–Si, respectively. The characteristic peak between 1,068 cm^−1^ is assigned to the asymmetric stretching vibration of Si–*O*–Si.^39^

The nitrogen adsorption isotherms and corresponding pore size distribution of Cu-MBG NPs are presented in Figure S2. The isotherms exhibited a typical type IV curve, with a distinct hysteresis loop, confirming the mesoporous nature of the material. The specific surface area and average pore size of the Cu-MBG NPs were calculated from the adsorption branch. The NPs exhibited a specific surface area of 13.8 m^2^/g and an average pore size of 13.3 Å (1.33 nm), further supporting their mesoporous structure.

### Synthesis and characterization of PU-Cu-MBG foam

**Figure 2:**
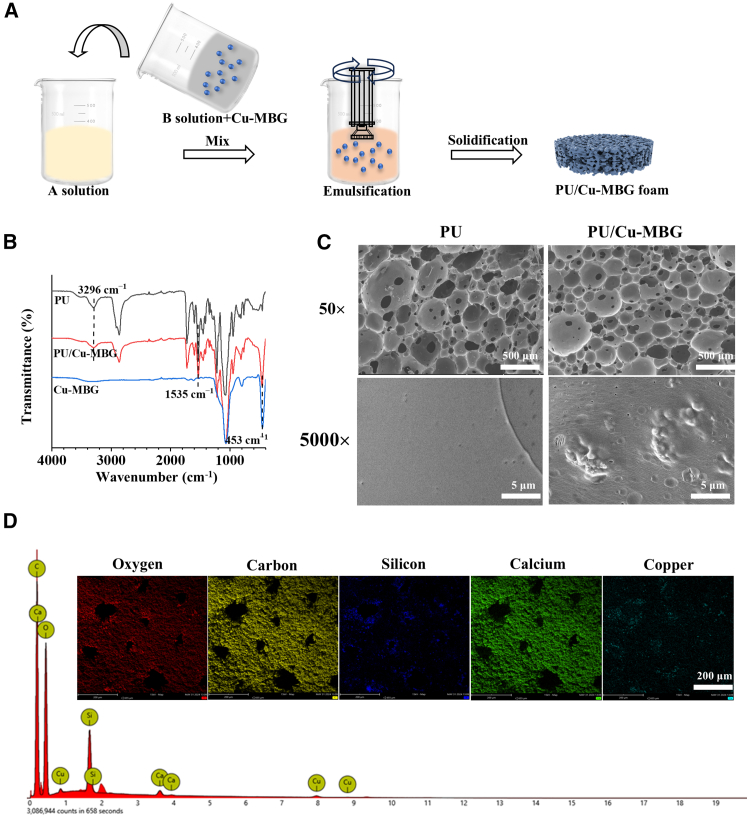
Synthesis and characterization of PU-Cu-MBG foam (A) Synthetic scheme of PU-Cu-MBG. (B) The FTIR spectra of PU, PU-Cu-MBG, and Cu-MBG. (C) SEM images of PU and PU-Cu-MBG in 50× (scale bars: 500 μm) and 5000× (scale bars: 5 μm) magnifications. (D) Mapping analysis images of PU-Cu-MBG (scale bars: 200 μm).

The preparation process of the composite foam is illustrated in Figure 2A. An aqueous solution containing the surfactant, foam stabilizer, and excipient was mixed with a certain amount of Cu-MBG NPs and vigorously stirred. Then, the solution was added to the PU prepolymer and stirred for 10 s, with the aid of an emulsifier, at 5,000 rpm. In this process, the residual –NCO group in the prepolymer and the –OH group of the water molecule react quickly to form urea group and produce a large amount of CO_2_. After curing and drying, the hydrophilic PU foam (PU and PU-Cu-MBG) was obtained. The chemical structure of the foam was verified using FTIR. As shown in Figure 2B, the absence of a characteristic –NCO peak at 2,260 cm^−1^ in the PU spectrum indicated that the –NCO groups in polyurethane prepolymer (PrePU) had fully reacted, suggesting that the material was non-cytotoxic. Additionally, the peaks near 3,296 and 1535 cm^−1^ are attributed to the stretching and bending vibrations of –NH in urethane and urea groups, respectively.^40^ In the spectrum of PU-Cu-MBG, the presence of vibration peaks (453 cm^−1^) corresponding to Cu-MBG confirmed that the inorganic NPs were successfully incorporated into the foam.

The porous structure of foam is one of the most crucial parameters in wound repair treatment. The morphology of PU and PU-Cu-MBG foams was studied, and SEM images at low magnification showed that both foams possessed excellent open-cell structures and characteristic features of PU soft foam. The pore size distribution was relatively uniform, with pore sizes ranging from 400 to 500 μm. Such a porous structure was conducive to absorbing wound exudate (Figure 2C). Additionally, energy-dispersive X-ray spectroscopy (EDS) element analysis was performed to study the distribution characteristics of Cu-MBG NPs on the foam (Figure 2D). The analysis showed that Si, calcium, and Cu elements were uniformly distributed throughout the foam skeleton, indicating that the NPs were well dispersed on the foam surface. The well-dispersed Cu-MBG on the PU foam surface ensured their efficient biological performance.

**Figure 3:**
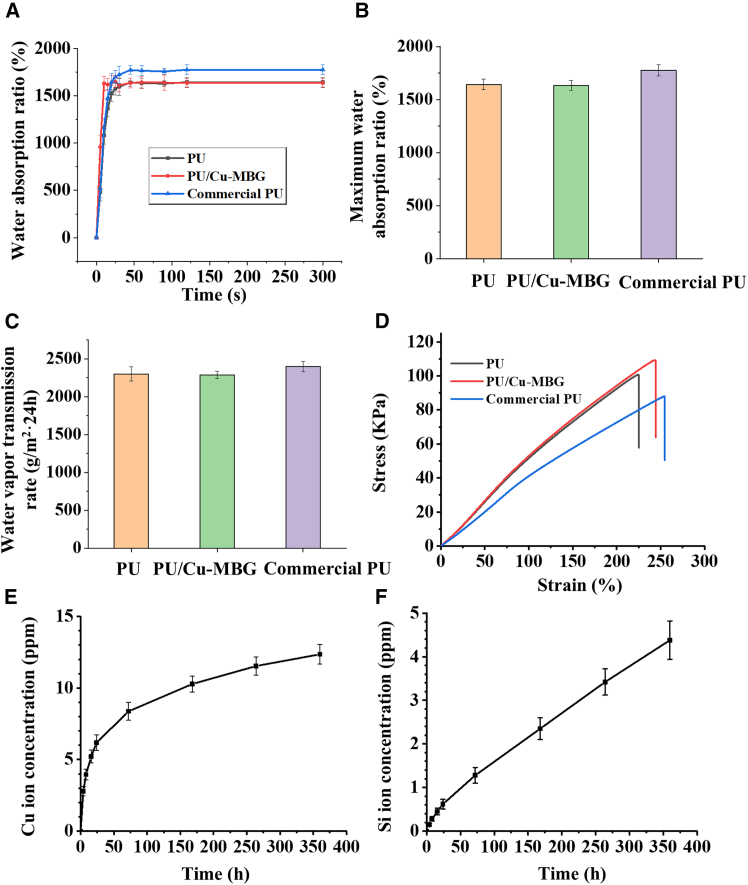
The physical properties of PU-Cu-MBG foam (A) The water absorption ratio of the three foams. (B) The maximum water absorption ratio of the three foams. (C) The water vapor transmission rate of the three foams. (D) The tensile stress-strain curves of the three foams. (E) Concentration of Cu^2+^ released from the foam after 15 days of immersion in PBS. (F) Concentration of Si^4+^ released from the foam after 15 days of immersion in PBS. All data in (A)–(F) are shown as mean ± SD (*n* = 3).

Next, the physical properties of the foams were further investigated. In addition to the two experimental foam groups, a commercially available PU foam (“commercial” PU) was also tested for comparison. Firstly, the liquid absorption capacity and water vapor transmission rate (WVTR) of the foams were evaluated. The results showed that both foams exhibited outstanding water absorption capacity, with the water absorption ratio exceeding 1600% within 50 s (Figure 3A). The maximum liquid absorption ratios of the PU, PU-Cu-MBG, and “commercial” PU foams were 1642.1% ± 51.2%, 1,634.7% ± 46.9%, and 1774.98% ± 50.92%, respectively (Figure 3B). The excellent liquid absorption performance of the foams is attributed to their physicochemical properties. Chemically, the hydrophilic groups on the surface of the PU foam facilitate the absorption of water molecules; physically, the large pore structure within the foam promotes the entry of water molecules. Besides, as shown in Figure 3C, the WVTR results indicated that the values of the three foams exceeded the threshold, ensuring that the foam dressings maintained a moist wound environment while effectively preventing excessive accumulation of exudates. Notably, these results demonstrated that the as-prepared foam exhibited comparable performance to the commercially available foam. Moreover, the incorporation of NPs did not significantly compromise the liquid absorption capacity of the foam, which may be attributed to the relatively low NP loading.

The mechanical stability of the foam throughout the healing process is crucial for providing full protection to the wound. Therefore, tensile stress-strain tests were performed (Figure 3D). As shown in Table 1, the elastic modulus of the PU and “commercial” PU foam was 50.38 ± 4.52 and 43.85 ± 2.97 KPa, the tensile strength was 110.53 ± 10.93 and 89.97 ± 5.12 KPa, and the elongation at break was 220.13% ± 4.47% and 259.04% ± 8.14%, respectively. For the PU-Cu-MBG foam, both the elastic modulus and tensile strength were slightly increased, while the elongation at break was improved. This further indicates that the introduction of NPs did not negatively impact the physical properties of the foam dressing.

**Table 1:** The tensile properties of two groups of foams

Sample	Elasticity modulus (KPa)	Tensile strength (KPa)	Elongation at break (%)
PU	50.38 ± 4.52	110.53 ± 10.93	220.13 ± 4.47
PU-Cu-MBG	51.99 ± 3.62	118.21 ± 10.43	237.18 ± 7.28
Commercial PU	43.85 ± 2.97	89.97 ± 5.12	259.04 ± 8.14

The release behavior of Cu^2+^ and Si^4+^ from the PU-Cu-MBG foam was evaluated in PBS (pH 7.4, 37°C) over 15 days, as shown in Figures 3E and 3F. Cu^2+^ exhibited a biphasic release pattern characterized by an initial burst release followed by sustained release. Within the first 24 h, the cumulative Cu^2+^ concentration reached 6.18 ± 0.54 ppm. Subsequently, the release continued gradually, attaining 12.36 ± 0.68 ppm by day 15. In contrast, Si^4+^ displayed extremely slow-release kinetics. The cumulative Si^4+^ concentration remained below 1 ppm during the first 24 h and could reached only 4.38 ± 0.35 ppm after 15 days, indicating the high stability of the silica framework.

### *In vitro* hemocompatibility, biocompatibility, and bioactivity

**Figure 4:**
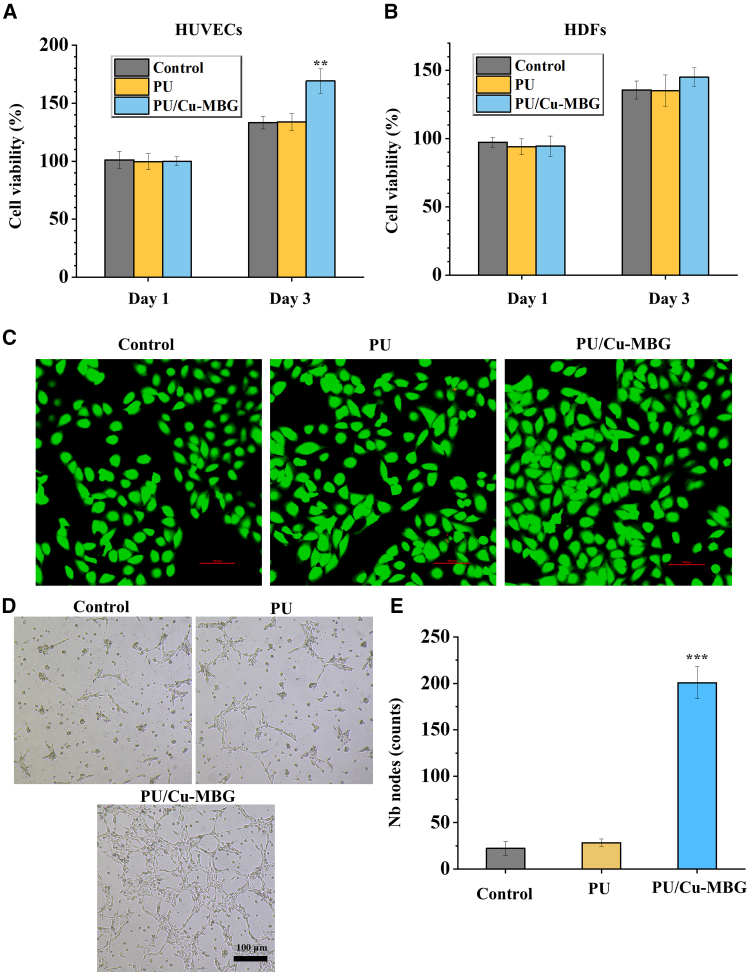
The bioactivity of PU-Cu-MBG foam (A) *In vitro* viability of HUVECs directly cultured with sample extracts. Data are presented as mean ± SD (*n* = 6). (B) *In vitro* cell viability of HDFs directly cultured with sample extracts. Data are presented as mean ± SD (*n* = 6). (C) Live/dead staining of HUVECs treated with different sample extracts for 72 h. Scale bars: 100 μm. (D) Angiogenesis images of HUVECs cultured on ECMatrix with different sample extracts for 6 h. Scale bars: 100 μm. (E) The Nb nodes in angiogenesis images was quantified using ImageJ. Data are presented as mean ± SD (*n* = 3). ∗∗*p* < 0.01, ∗∗∗*p* < 0.001.

For wound healing applications, the biosafety of the dressing materials is one of the critical issues to be addressed. Firstly, the hemocompatibility of PU and PU-Cu-MBG was evaluated using a hemolysis assay. As shown in Figure S3, the hemolysis ratios of PU and PU-Cu-MBG were 2.42% ± 0.43% and 1.64% ± 0.34%, respectively, both of which are below the 5% threshold set by national standards. Next, the cytotoxicity of the foam was evaluated by CCK-8 method. As shown in the Figures 4A and 4B, the extracts of the foam (1 mg/mL) had no obvious cytotoxicity (˂75%) to human umbilical vein endothelial cells (HUVECs) and human dermal fibroblasts (HDFs) after incubation for 1 day. The extracts of the composite foam even exhibited bioactivity to stimulate the proliferation of HUVECs after incubation for 3 days as compared with that of the blank control and PU group. Besides, as shown in Figure 4C, a similar conclusion to the CCK-8 assay was drawn from the live/dead staining results, in which the number of PU-Cu-MBG group cells was significantly higher than those in the blank control group and the PU group. Furthermore, as shown in Figures 4D and 4E, HUVECs treated with extracts from PU-Cu-MBG foam formed significantly more tube-like structures, including increased tube length, branching points, and mesh-like networks, compared with those in the control and PU groups.

The biological activity of the composite foam can be attributed to the active components, Cu-MBG, loaded within it. It has been reported that trace amounts of Si ions released from Cu-MBG promote the proliferation and migration of fibroblasts and endothelial cells.^34^ Additionally, the released Cu ions affect cells in a dose-dependent manner. At lower amounts, Cu^2+^ modulate cellular signaling pathways and stimulate fibroblast growth in the skin. Conversely, at high levels exceeding the body’s requirements, Cu^2+^ may be cytotoxic, participating in reactions that produce highly reactive oxygen species (ROS), which are responsible for lipid peroxidation in membranes, direct oxidation of proteins, and degradation of DNA and RNA.^32^

### *In vitro* antibacterial property

**Figure 5:**
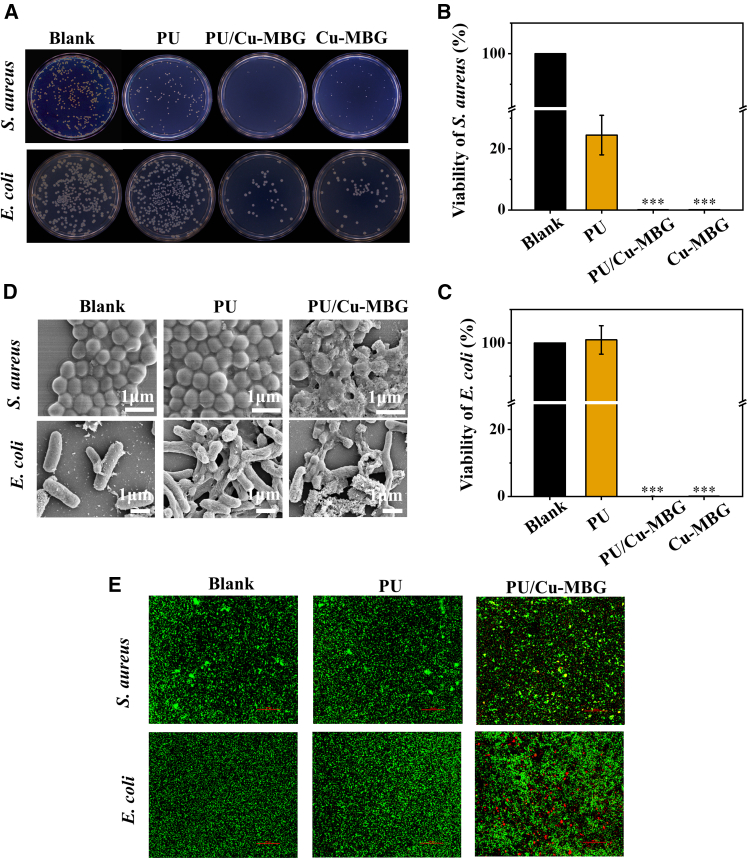
Antibacterial property of all samples (A) Representative images showing the survival of bacteria in the culture plate. (B) Quantitative antibacterial test results for different samples of *S. aureus*. Data are presented as mean ± SD (*n* = 3). (C) Quantitative antibacterial test results for different samples of *E. coli*. Data are presented as mean ± SD (*n* = 3). (D) SEM images of *S. aureus* and *E. coli* with different samples. Scale bars: 1 μm. (E) The images of live/dead staining for *S. aureus* and *E. coli* treated with different samples. Scale bars: 100 μm. ∗∗∗*p* < 0.001.

The antibacterial properties of the two foams and Cu-MBG against *S. aureus* and *E. coli* were evaluated using the agar plate method (Figures 5A–5C). After co-culturing bacteria with the materials, we found that the bacterial count in the PU group remained largely unchanged compared with the control group, indicating that the conventional PU foam lacks antibacterial properties. Cu-MBG exhibited excellent antibacterial performance against both types of bacteria, and when combined with the foam dressing, it retained its antibacterial properties. The bacterial quantity significantly decreased in the PU-Cu-MBG group, with almost no bacterial growth observed. Statistical analysis confirmed that Cu-MBG and PU-Cu-MBG foam demonstrated a significant antibacterial effect against Gram-positive *S. aureus* and Gram-negative *E. coli*, with the bacterial viability of Cu-MBG NPs and PU-Cu-MBG foam being nearly zero. It is worth noting that PU also exhibited some antibacterial activity against *S. aureus*, which may be attributed to the action of surfactants incorporated within it against Gram-positive bacteria.

Next, the morphologies and membrane integrities of *S. aureus* and *E. coli* were examined by a scanning electron microscope. As shown in Figure 5D, the morphologies of *S. aureus* and *E. coli* in the blank group showed a smooth morphology. The bacteria in the PU group also exhibited a similar morphology. In contrast, obvious membrane collapse and deformation were observed for *S. aureus* and *E. coli* in the PU-Cu-MBG group. Additionally, live/dead staining followed by confocal laser scanning microscopy (CLSM) analysis was used to evaluate the antibacterial activity against *S. aureus* and *E. coli.* As shown in the corresponding fluorescence images of *S. aureus* and *E. coli* (Figure 5E), while the blank and PU groups exhibited almost exclusively green staining for both bacteria, red and yellow fluorescent clusters (the overlay of red and green) appeared in the PU-Cu-MBG group. This indicated that the composite foam effectively killed a proportion of bacterial cells.

On the basis of this analysis, we concluded that the antibacterial properties of the dressing are primarily related to the Cu^2+^ released from Cu-MBG. The antibacterial characteristics of Cu^2+^ may involve multiple mechanisms, such as internalization within cells, electrostatic interactions with microorganisms, prevention of DNA replication, reduction of enzyme activity, and generation of ROS.^41^ In addition to its rapid antibacterial effect within the first 24 h, the sustained release profile of Cu^2+^ from the PU-Cu-MBG foam suggests the potential for prolonged antibacterial activity. Based on the aforementioned Cu^2+^ release study, the cumulative release of Cu^2+^ reached 6.18 ± 0.54 ppm at 24 h and continued to increase gradually, attaining 12.36 ± 0.68 ppm by day 15. This sustained release behavior indicates that the foam dressing is capable of continuously supplying bioactive Cu^2+^ over an extended period, which may help maintain a local environment unfavorable for bacterial re-colonization during the wound healing process.^34^^,^^42^ Therefore, the PU-Cu-MBG foam not only provides rapid initial bacterial inhibition but also offers prolonged ion release that could contribute to long-term protection against infection in chronic wounds.

### Treatment of diabetic wound

**Figure 6:**
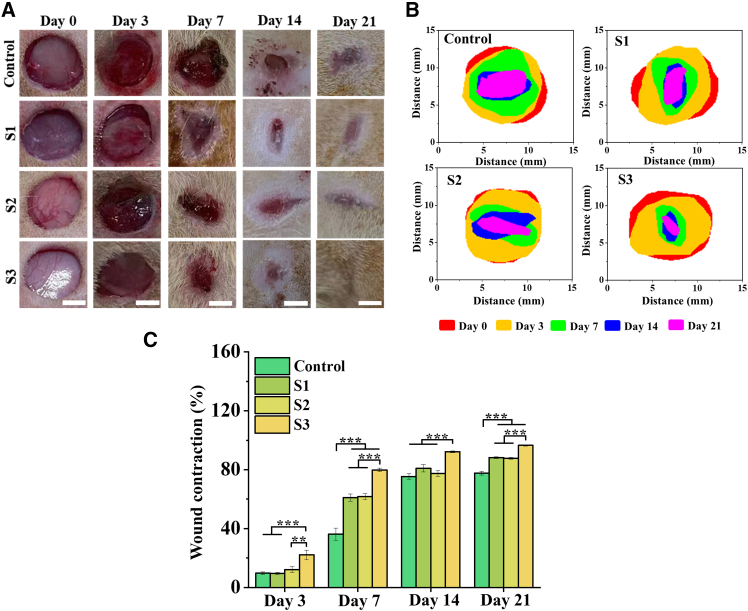
Diabetic SD rat full-thickness skin defect wound healing assessment (A) Representative images of skin wound treated with different samples (S1, commercial foam; S2, PU foam; S3, PU-Cu-MBG). Scale bars: 5 mm. (B) The wound traces of different samples at 0, 3, 7, 14, and 21 days. (C) The corresponding wound contraction rates of different samples at 0, 3, 7, 14, and 21 days. Data are expressed as mean ± SD. *n* = 5; ∗∗*p* < 0.01, ∗∗∗*p* < 0.001.

Chronic wounds are a major health issue worldwide, with diabetic chronic wounds being a typical example. It has been reported that maintaining a moist wound environment and promoting angiogenesis are two crucial factors influencing wound healing.^43^ Additionally, high glucose levels provide an optimal environment for the proliferation of pathogenic bacteria, leading to prolonged wound infections.^44^ Therefore, antibacterial treatment for wounds is also a key factor to consider. To this end, a model of diabetic skin wound defects in SD rats was constructed to verify the feasibility of using composite foam dressings for wound repair. First, a type 2 diabetic rat model was established by feeding the rats a high-sugar, high-fat diet and administering intraperitoneal injections of streptozotocin (STZ) solution. After confirming the diabetic status through blood glucose testing, the rats were divided into four groups (Figure S4). Defect wounds were created on the skin of the rats, and the wound healing status was observed on days 3, 7, 14, and 21. As shown in Figure 6A, the wound area in all groups gradually decreased with the increase of days, and the wounds in the PU-Cu-MBG group were fully healed within 21 days. The PU-Cu-MBG group exhibited significantly better healing effect than the commercial foam group and the PU group. After 7 days, the wound contraction rates of the blank control, commercial foam, PU, and PU-Cu-MBG groups were 36.22% ± 4.14%, 61.05% ± 2.52%, 61.72% ± 1.97%, and 79.80% ± 1.16%, respectively. Compared with the blank control group, both the commercial and synthesized foams accelerated the early-stage wound healing. Moreover, the composite foam dressings further promoted wound repair compared with the blank foam. The wound contraction rate of the PU-Cu-MBG group reached 96.56% ± 0.49% after 21 days, while those of the control, commercial foam, and PU groups were 77.71% ± 1.28%, 88.20% ± 0.63%, and 87.74% ± 0.64%, respectively (Figures 6B and 6C). From the above results, it could be seen that using conventional foam dressings, which absorbed tissue exudate and acted as a barrier to bacteria, could accelerate the healing of diabetic wounds. Further combining these foam dressings with the antibacterial properties and bio-activity of Cu-MBG significantly enhanced the wound healing process.

**Figure 7:**
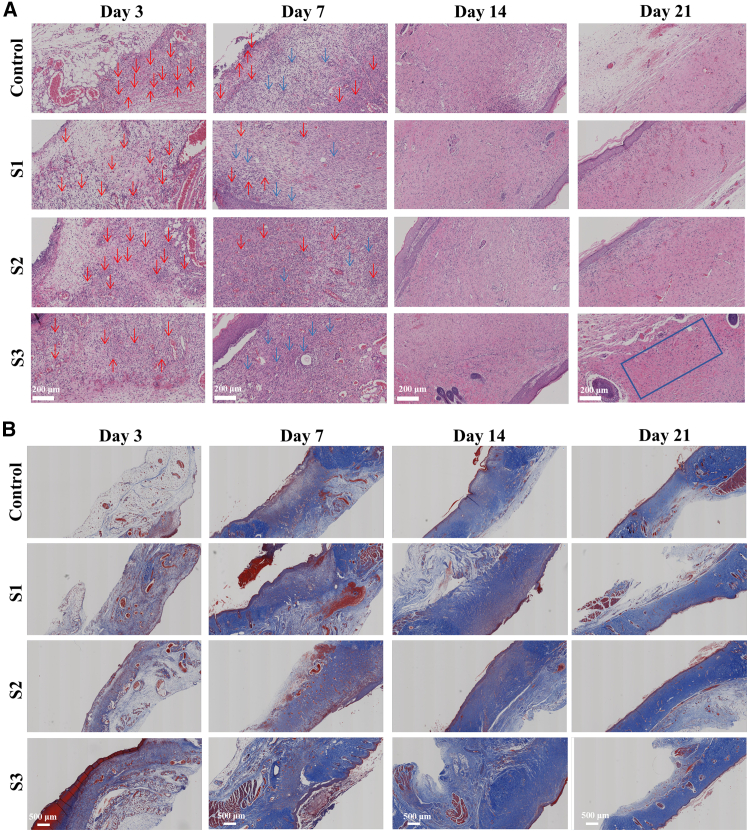
Results of H&E staining and masson trichrome staining (A) H&E staining evaluation of wound healing for different samples (S1, commercial foam; S2, PU foam; S3, PU-Cu-MBG) after 3, 7, 14, and 21 days. *n* = 5. Red arrows, inflammatory cells; blue arrows, fibroblasts; blue box, granulation tissue. Scale bars: 200 μm. (B) Masson trichrome staining evaluation of wound healing for different samples (S1: commercial foam; S2: PU foam; S3: PU-Cu-MBG) after 3, 7, 14, and 21 days (*n* = 5). Scale bars: 500 μm.

To further evaluate the wound healing effects of different materials, we used H&E staining and Masson’s trichrome staining (Figures 7A, 7B, S5, and S6). At day 3, all groups showed noticeable inflammatory cell aggregation. However, in contrast to the blank group, the three material groups exhibited slight collagen deposition. At day 7, in the blank control, commercial foam, and PU groups, aggregation of inflammatory cells and some fibroblasts could be observed. However, in the PU-Cu-MBG group, inflammatory cells were nearly absent, but a large number of fibroblasts were present. Additionally, the Masson’s staining results showed that the PU-Cu-MBG group exhibited more collagen deposition. At day 14, the collagen density in the wound repair areas of different groups gradually increased, and collagen deposition in the PU-Cu-MBG group was higher than that in the other groups. Besides, immunohistochemical staining for CD31 was performed on the wound tissue sections. As presented in Figure S7, the PU-Cu-MBG group exhibited significantly higher CD31-positive microvessel density than the control and PU groups at day 14 post-wounding. At last, compared with the other groups, clear new blood vessels, organized granulation tissue, and hair follicle structures could be observed in the PU-Cu-MBG group.

Wound healing encompasses a complex process of hemostasis, inflammation or migration, proliferation, and tissue remodeling. For chronic wounds, the healing process is often hindered by excessive tissue exudate, bacterial invasion, and impaired angiogenesis. Foam dressings, due to their 3D porous structure and hydrophilicity, facilitate the absorption of wound exudate, creating a moist environment that accelerates wound healing. Building on this, the Cu-MBG incorporated within the dressing leverages the bioactive properties of its released Cu^2+^. Beyond its direct antibacterial effect, it is well documented that Cu^2+^ stimulate the expression of angiogenic factors such as VEGF and FGF. This process is primarily mediated by the activation of multiple signaling cascades, including the hypoxia-induced VEGF secretion pathway, as well as the MAPK and tyrosine kinase pathways.^31^ This stimulation of VEGF, in turn, enhances endothelial cell proliferation and migration, leading to accelerated angiogenesis. Furthermore, Cu^2+^ play a crucial role in the wound healing cascade by promoting the proliferation and migration of fibroblasts and enhancing the maturation of collagen.^45^^,^^46^ Consequently, the multifunctional Cu-MBG dressing not only eliminates bacteria in the wound tissue but also actively orchestrates cellular responses, promoting cell proliferation, angiogenesis, and collagen deposition to significantly accelerate the healing process.

## Discussion

PU foam dressings can effectively protect chronic wounds, reduce secondary damage, absorb wound exudate, and maintain a moist wound environment, thereby decreasing the frequency of drug changes. Additionally, some functional foam dressings can slowly release antibiotics, silver ions, and growth factors, continuously acting on the wound to accelerate the healing process. For instance, Chen et al. developed a modified PU foam dressing with high fluid absorption capacity, which, in a diabetic rat wound model, shortened the inflammatory phase and enhanced collagen deposition, accelerating the wound repair process.^19^ Li et al. prepared a lignin PU/silver composite foam with improved mechanical, thermal, and antibacterial properties. This composite foam could effectively promote wound healing in full-thickness skin defects in mice.^22^ However, chronic wound healing is a complex process, and relying solely on the physical properties of foam dressings or conventional slow-release treatments with antibiotics and silver ions is insufficient. Therefore, there is an urgent need to modify foam dressings to endow them with greater bioactivity.

Bioactive glass and its organic composites have recently been shown to promote not only hard tissue regeneration but also the regeneration of various soft tissues, including muscle, tendon, and skin. Among these, Cu-MBG exhibits broad-spectrum antibacterial properties due to its ability to release Cu^2+^. Furthermore, the synergy between Si^4+^and Cu^2+^ can produce a stronger angiogenic effect, accelerating the repair of chronic wounds. We propose that loading these materials into foam dressings may synergistically enhance the regulation of chronic wound repair through the combined physical action of the dressing and the biological efficacy of the NPs. However, there are two key issues that deserve focused research in the preparation of organic/inorganic composite materials. One is how to effectively load inorganic NPs into foams with a 3D porous structure, and the other is whether the interaction between the two materials affects their overall physical and biological properties.

To better utilize the bioactivity of MBG, researchers have extensively developed inorganic-polymer nanocomposite materials such as sponges, fibers, and hydrogels. Typically, NPs are mixed with polymer solutions before being fabricated into various forms of materials. In this work, to enhance the effective loading of Cu-MBG in foam, we added sodium carboxymethyl cellulose (CMC) to the reaction system. This was intended to stabilize the distribution of Cu-MBG in the mixed aqueous solution (B solution) through hydrogen bonding between CMC and Cu-MBG before reaction with the PU prepolymer, ultimately achieving effective loading of Cu-MBG in the foam dressing during the polymerization process. SEM images and mapping analysis results indicate that Cu-MBG is not simply mixed but is encapsulated within the polymer matrix and uniformly distributed throughout the foam, reducing excessive leakage of the active material during subsequent use.

Next, we investigated the physical and biological properties of the composite foam dressing. The results showed that the incorporation of Cu-MBG did not affect the foam’s water absorption ratio or WVTR. This is attributed to the uniform distribution of Cu-MBG within the foam structure, ensuring the connectivity of the foam’s porous structure. Furthermore, the PU-Cu-MBG composite exhibited better mechanical performance than PU. This improvement may be due to Cu-MBG acting as a solid filler and the formation of additional hydrogen bonds between Cu-MBG and the polymer, which enhanced the foam’s structural integrity. From the bioactivity results, we conclude that the effective loading of Cu-MBG allowed the composite foam to both stimulate the proliferation of HUVECs and demonstrate inherent antibacterial effects against Gram-positive *S. aureus* and Gram-negative *E. coli*. These results indicate that the encapsulation of Cu-MBG by the polymer did not restrict its bioactivity; Si^4+^, Ca^2+^, and Cu^2+^ could still be released from the foam dressing.

*In vivo*, the PU-Cu-MBG foam significantly accelerated diabetic wound healing compared with commercial and pure PU foam controls. This was achieved through the inhibition of excessive inflammation, promotion of fibroblast proliferation, enhanced angiogenesis, and increased collagen deposition.

In summary, a multifunctional composite foam dressing (PU-Cu-MBG) was successfully developed by incorporating Cu-MBG NPs into a PU matrix. The resulting dressing exhibited excellent liquid absorption, robust mechanical properties, and sustained release of bioactive ions, enabling both antibacterial activity and pro-angiogenic effects. In a diabetic rat wound model, PU-Cu-MBG significantly accelerated healing by modulating inflammation, promoting angiogenesis, and enhancing collagen deposition. With its integrated physical and biological functions, this composite foam represents a promising candidate for the treatment of chronic diabetic wounds.

### Limitations of the study

This study has several limitations. First, the long-term biosafety and degradation behavior of the composite foam *in vivo* require further investigation. Second, while the synergistic effects of released ions were observed, the precise molecular mechanisms underlying their pro-angiogenic and anti-inflammatory activities remain to be elucidated. Third, the current fabrication method may need optimization for large-scale production, and batch-to-batch variability in terms of NP distribution, mechanical properties, and bioactivity should be systematically evaluated to ensure reproducibility and consistency of the composite foam dressing.

## Resource availability

### Lead contact

Requests for further information, resources, and data should be directed to and will be fulfilled by the lead contact, Yili Xiang (xiangmily@126.com).

### Materials availability

All unique/stable reagents generated in this study are available from the lead contact, with a completed material(s) transfer agreement.

### Data and code availability

Data: All data reported in this article are available from the lead contact upon request.
Code: This paper does not report original code.
All other items: Any additional information required to reanalyze the data reported in this paper is available from the lead contact upon request.

## Acknowledgments

This work was financially supported by the Outstanding Young Talents of Clinical Research Talents of Wenzhou People’s Hospital (RC-PH202241(QN)) and the Wenzhou Scientific Research Project Fund (Y20240711).

## Author contributions

Conceptualization, Y.X.; data curation, L.X., D.H., K.C., B.Z., and J.L.; methodology, L.X., D.H., K.C., B.Z., and J.L.; investigation, L.X., D.H., K.C., and Y.Z.; validation, L.X., D.H., K.C., and Y.Z.; writing – original draft, L.X.; writing – review & editing, Y.X.; supervision, S.W. and M.W.; project administration, Y.X.; funding acquisition, Y.X.

## Declaration of interests

The authors declare no competing interests.

## STAR★Methods

### Key resources table

**Table:** 

REAGENT or RESOURCE	SOURCE	IDENTIFIER
**Antibodies**		

Anti-CD31 Mouse	Servicebio	Cat# GB12063-100；RRID：AB_2941868
HRP conjugated Goat Anti-Mouse IgG (H + L)	Servicebio	Cat# GB23301；RRID：AB_2904020

**Bacterial and virus strains**		

S. aureus	China General Microbiological Culture Collection Center	ATCC 25923
E. coli	China General Microbiological Culture Collection Center	ATCC 25922

**Chemicals, peptides, and recombinant proteins**		

Cetyltrimethylammonium bromide	Shanghai Macklin Biochemical Co., Ltd	Cat# H811115-100 g
Anhydrous ethanol	Shanghai Macklin Biochemical Co., Ltd	Cat# E809056-500 mL
Ethyl acetate	Shanghai Macklin Biochemical Co., Ltd	Cat# E809174-500 mL
Ammonia solution	Shanghai Macklin Biochemical Co., Ltd	Cat# A834475-500 mL
Tetraethyl orthosilicate	Shanghai Macklin Biochemical Co., Ltd	Cat# T819505-100 mL
Ca(NO_3_)_2_·4H_2_O	Shanghai Macklin Biochemical Co., Ltd	–
CuCl_2_·2H_2_O	Shanghai Macklin Biochemical Co., Ltd	Cat# C805298-100 g
Pluronic L45	Shanghai Aladdin Biochemical Technology Co., Ltd	Cat# S434418-250 g
Carboxymethyl cellulose	Shanghai Aladdin Biochemical Technology Co., Ltd	Cat# C104981-100 g
Silicone oil L580	Momentive Performance Materials Inc	Cat# DT2827E9827
DMEM	Thermo Fisher	Cat# C11995500BT

**Critical commercial assays**		

CCK-8 kit	Dojindo	Cat#CK04-5000 T
SYTO 9/PI Live/Dead Bacterial Viability Kit	Thermo Fisher	Cat# L7012
Hematoxylin and Eosin (H&E) High-Definition Permanent Staining Kit	Servicebio	Cat# G1076-500 ML
Masson trichrome staining Kit	Servicebio	Cat# G1006-100 ML

**Experimental models: Cell lines**		

HUVECs	Wuhan Pricella Biotechnology Co., Ltd.	Cat# CP-H082
HDFs	Wuhan Pricella Biotechnology Co., Ltd.	Cat# CP-H103

**Experimental models: Organisms/strains**		

SD rats	Zhejiang Laboratory Animal Center	Cat#19079
Rabbit	Zhejiang Laboratory Animal Center	Cat#21461

**Software and algorithms**		

IBM SPSS Statistics 20.0	IBM Corp	https://www.ibm.com/products/spss-statistics
ImageJ	National Institutes of Health (NIH)	https://imagej.nih.gov/ij/
Origin	OriginLab Corporation	https://www.originlab.com

### Experimental model and study participant details

#### Animal experiment

All animal experiments were performed in strict accordance with National Institutes of Health (NIH) guidelines for the care and use of laboratory animals and approved by Wenzhou Institute of UCAS. Ethics approval number: WIUCAS26022603. The male Sprague Dawley (SD) rats (200–250 g), purchased from the Zhejiang Laboratory Animal Center, were fed a high-sugar, high-fat diet for 4 weeks. After this period, they received intraperitoneal injections of STZ for three consecutive days. Seventy-two hours after the final STZ injection, fasting blood glucose levels were measured. Rats with blood glucose levels above 14 mmol/L were considered successfully modeled. The successfully modeled rats were then randomly divided into four groups. Next, the *in vivo* wound healing experiments were carried out by a full-thickness skin defect model. The rat was anesthetized by intramuscular injection of quantitative Zoletil 50. The full thickness skin wound was made by a biopsy punch (diameter = 10 mm). Then, the foams were applied on the wounds and adhered by Tegaderm™ film. On different time intervals (3, 7, 14 and 21 d), the process of wound healing was observed and photographed. The skin tissues were collected for further analysis.

#### Cell lines

The Human Umbilical Vein Endothelial Cells (HUVECs) and Human Dermal Fibroblasts (HDFs) were purchased from Wuhan Pricella Biotechnology Co., Ltd. (Cat. No. CP-H082 and CP-H103).

#### Microbe strains

The bacterial strains used in this study included *Staphylococcus aureus* (ATCC 25923) and *Escherichia coli* (ATCC 25922), both obtained from the China General Microbiological Culture Collection Center (CGMCC).

### Method details

#### Synthesis and characterization of Cu-MBG NPs

Cu-MBG NPs were synthesized using the sol-gel method. First, 0.5 g of CTAB was dissolved in 50 mL of deionized water. Once completely dissolved, 8 mL of ethyl acetate was added and the mixture was stirred magnetically for 0.5 h. Next, 5 mL of NH_3_·H_2_O was added, and after stirring for another 0.5 h, TEOS and Ca(NO_3_)_2_·4H_2_O were added sequentially. Subsequently, a measured amount of CuCl_2_·2H_2_O was added to the mixture, and the reaction was allowed to proceed for an additional 4 h. The resulting product was collected by centrifugation at 10,000 rpm for 10 min and washed three times with ethanol, followed by three additional washes with deionized water to ensure the complete removal of residual CTAB and other additives. After washing, the product was dried at a constant temperature of 60°C for 12 h. The obtained powder was then calcined in a muffle furnace, with the temperature increased at a rate of 2°C/min to 700°C and maintained for 4 h, resulting in the formation of Cu-MBG NPs.

The microstructure and element distribution of Cu-MBG NPs were characterized by scanning electron microscopy (SEM) equipped with an energy-dispersive X-ray (EDX) detector for compositional analysis. The size distribution and surface zeta potential of the nanoparticles were detected by dynamic light scattering (DLS, Zetasizer Nano ZS ZEN3600) The chemical composition of the four-group particles was measured using EDX analysis during SEM observation. X-ray diffraction analysis (XRD) was conducted in a 2θ range of 20–80°. XPS measurements were carried out to determine the surface functionalization of Cu-MBG NPs and mean oxidation state of Cu. N_2_ adsorption isotherms were measured using a surface area and porosity analyzer to determine the specific surface area and pore size distribution of the samples. Prior to analysis, the samples were degassed at 220°C.

#### Synthesis of PU/Cu-MBG composite foam

Pluronic L45 (15 wt%), silicone oil L580 (5 wt%), CMC (5 wt%) and Cu-MBG NPs (5 wt%) were dispersed in distilled water to obtain an aqueous mixture. Then, at 20°C, the aqueous polyurethane prepolymer was added to the aqueous mixture at a mass ratio of 1:1 and stirred vigorously for 10 seconds using an emulsifying mixer with a shear blade. After foaming, the unstable foam solution was placed in an oven at 80°C for curing and drying. This process yielded PU/Cu-MBG composite foam. Control PU foam was prepared similarly, except without the addition of NPs to the aqueous mixture.

#### Characterization of foam

The chemical structure of PU and PU/Cu-MBG was confirmed by FT-IR (Magna-560, Nicolet). All samples for FT-IR were characterized by attenuated total reflectance (ATR) method. The microstructure of foam under different magnification were observed by scanning electronic microscopy (SEM, SU8010, HITACHI, Japan) and EDX mapping analysis were also collected. The water absorption ratio of foam was measured by previously reported methods. The foam was weighed (*W*_*0*_) before test and then immersed into distilled water for certain time. After that, the sponges were taken out and lightly dabbed on filter papers to remove the excess water, the weight of final sponges were recorded as *W*_*1*_. The water absorption ratio was calculated from the following equation: (*W*_*1*_*-W*_*0*_)*/ W*_*0*_×100%. The mechanical properties of foam were measured by a UTM2102 electronic universal testing machine. Under room-temperature, the rectangle foam (40 mm (length) × 20 mm (width) × 10 mm (thickness)) was stretched with a speed of 10 mm/min. Each group was repeated for three times. The release behavior of Cu^2+^ and Si^4+^ in foam was determined by inductively coupled plasma optical emission spectrometry (ICP-OES, 7850, Agilent). 65 mg of the foam was immersed in 10 mL of phosphate-buffered saline (PBS, pH 7.4) and incubated at 37°C under gentle shaking (100 rpm). At predetermined time intervals (0 h, 4 h, 8 h, 16 h, 24 h, 3 d, 7 d, 11 d, and 15 d), 1 mL of the release medium was collected and replaced with an equal volume of fresh pre-warmed PBS. The concentrations of Cu^2+^ and Si^4+^ in the medium were measured. All experiments were performed in triplicate.

#### *In vitro* cell culture experiments of the foam

Human umbilical vein endothelial cells (HUVECs) and human dermal fibroblasts (HDFs) were selected for evaluating the biological effects of the foam in this study. Before test, the extract of foam was prepared. 0.1 g of foam was soaked in 10 mL serum-free culture medium and incubated at 37°C for 24 h. Then, the supernatant was sterilized through a filter membrane. HUVECs and HDFs were seeded on 96-well plates at 1 × 10^3^ cells, respectively, and cultured with a 100 μL extract. After being cultured for 24 h and 72 h, CCK-8 solution was added into each well for another 2 h of incubation. At last, the OD value (both the experimental groups and the blank groups) of the incubated solution was tested by a Microplate Reader(Varioskan LUX, ThermoFisher)at 450 nm.

Following the previously described grouping and treatments, the extracted sample solutions were incubated with HUVECs for 72 h. Subsequently, fluorescein diacetate was added to each well, and cell morphology was observed under a confocal laser scanning microscope (CLSM) with excitation at 490 nm. Representative images were captured for each group. Cells without any treatment were used as the blank control.

To determine the *in vitro* angiogenesis of the foams, HUVECs (3 × 10^4^ cells per well) were cultured on ECMatrix (Millipore) with previously described extract. At 6 h, photos were taken from five random microscopic fields using an inverted light microscope.

#### *In vitro* hemolysis ratio of the foam

Fresh blood was anticoagulated with sodium citrate and subsequently diluted tenfold using physiological saline. The prepared samples were then incubated with the diluted blood at 37°C for 6 hours. Following incubation, the supernatant was collected and its optical density (OD) was measured at 540 nm. For comparative purposes, whole blood diluted tenfold with deionized water served as the positive control, while whole blood diluted tenfold with physiological saline served as the negative control. The hemolysis ratio (H%) was calculated according to the following formula:

$$H\left(\%\right)=\frac{OD_{sample}-OD_{negative\;control}}{OD_{\;positive\;control}-OD_{negative\;control}}$$

where OD_sample_, OD_positive control_, and OD_negative control_ represent the OD values at 540 nm for the sample, positive control, and negative control, respectively.

#### *In vitro* antibacterial test of the foam

The antimicrobial property of foam was performed by colony counting. Gram-positive bacteria *S. aureus* and Gram-negative bacteria *E. coli* were used as the model microorganisms. 100 mg of foam was incubated with 900 μl sterilized PBS and 100 μl bacteria solution with the concentration of 5×10^5^ CFU/ml at 37°C for 4 h. Afterwards, 100 μl bacterial suspension was incubation for 24 h on 1.5% LB agar plates and the total number of colonies was recorded. The bacteria solution treated without sample was set as blank control group. Three replicates were conducted for each group and the survival ratio of bacteria were determined.

The bacterial morphology was examined using scanning electron microscopy (SEM). Briefly, *S. aureus* and *E. coli* suspensions (10^8^ CFU/mL, 1 mL) were incubated with 100 mg of foam saturated with the sterilized PBS at 37°C for 24 h. The bacterial cells were then collected, washed with PBS, and fixed with 2.5% glutaraldehyde solution for 24 hours. Subsequently, the fixed cells were dehydrated through a graded ethanol series (30%, 50%, 70%, 80%, 90%, and 100% v/v), followed by air-drying and gold sputtering prior to SEM observation.

Following incubation of *S. aureus* and *E. coli* suspensions (10^8^ CFU/mL, 1 mL) with 100 mg of foam saturated with the sterilized PBS at 37°C for 24 h, the bacterial cells were harvested and washed thrice with PBS via centrifugation. Prior to imaging with a confocal laser scanning microscope (CLSM), the cells were stained for 30 minutes using a SYTO 9/PI Live/Dead Bacterial Viability Kit (Thermo Fisher, L7012).

#### Wound contraction

The initial wound area (*A*_*0*_) and wound area in Day 3, 7, 14, 21 (*A*_*n*_) were measured by Image J. Wound contraction (%) was calculated by the following formula:

$$\text{Wound}\;\text{contraction}\;\left(\%\right)=\frac{A_{0}-A_{n}}{A_{0}}\times 100\%$$

#### H&E and masson staining

On different time intervals, the skin tissues from the wound area were harvested and fixed in 4% paraformaldehyde for 24 h at room temperature. The tissues were subsequently dehydrated through a graded ethanol series, cleared in xylene, embedded in paraffin, and sectioned into 5 μm-thick slices.

For H&E staining, the paraffin sections were first deparaffinized in xylene and rehydrated with decreasing concentrations of ethanol. The sections were then stained with hematoxylin for 3-5 min and rinsed with running water, followed by differentiation in hydrochloric acid alcohol and bluing treatment. Subsequently, the sections were counterstained with eosin for 1-2 min, dehydrated through graded ethanol, cleared with xylene, and mounted with neutral resin. H&E staining was used to evaluate inflammatory cell infiltration, re-epithelialization, and overall tissue morphology.

For Masson’s trichrome staining, the deparaffinized and rehydrated sections were treated according to the manufacturer’s instructions. Briefly, the sections were stained sequentially with hematoxylin, acid fuchsin-ponceau solution, and phosphomolybdic acid solution, followed by staining with aniline blue to visualize collagen fibers. After differentiation with weak acid solution, the sections were dehydrated, cleared, and mounted. Masson staining was used to assess collagen deposition and tissue remodeling in the wound area.

Finally, the stained tissue sections were observed and photographed under an optical microscope.

#### Immunohistochemical analysis

After 14 days of treatment, rats were euthanized and the skin tissues from the wound area were harvested. The collected tissues were fixed in 4% paraformaldehyde for 24 h at room temperature, followed by dehydration through a graded ethanol series, paraffin embedding, and sectioning into 4-5 μm thick slices. The tissue sections were deparaffinized in xylene and rehydrated with decreasing concentrations of ethanol. Antigen retrieval was performed by heating the sections in citrate buffer (pH 6.0) using a microwave oven. Endogenous peroxidase activity was blocked with 3% hydrogen peroxide for 10 min, and nonspecific binding sites were blocked with 5% bovine serum albumin (BSA) for 30 min at room temperature.

Subsequently, the sections were incubated overnight at 4°C with primary antibody against rat CD31. After washing with phosphate-buffered saline (PBS), the sections were incubated with the corresponding HRP-conjugated secondary antibody for 1 h at room temperature. The immunoreactive signals were visualized using 3,3′-diaminobenzidine (DAB) chromogen, and the nuclei were counterstained with hematoxylin. Finally, the stained sections were dehydrated, mounted, and observed under an optical microscope. Images were captured from representative fields, and the expression levels of CD31 were quantitatively analyzed using ImageJ software.

### Quantification and statistical analysis

All experiments were performed independently at least three times (n ≥ 3), and data are expressed as mean ± standard deviation (SD). Statistical analyses were conducted using IBM SPSS Statistics 20.0 software. Comparisons among multiple groups were evaluated by one-way analysis of variance (ANOVA) followed by Tukey's post-hoc test. When the assumptions of ANOVA were violated, the nonparametric Kruskal-Wallis test with Dunn's multiple comparisons test was employed. Statistical significance was set at ∗p < 0.05, ∗∗p < 0.01, and ∗∗∗p < 0.001.
