# Supplementary material for: Fabricating a Cu-MBG-incorporated polyurethane foam with antibacterial properties and bioactivity for diabetic wound healing
Source: iScience. 2026 Jun 17;29(7):116423. doi: 10.1016/j.isci.2026.116423 (PMC13293721; doi:10.1016/j.isci.2026.116423)
Supplement: Document S1. Figures S1–S7 [file mmc1.pdf]

iScience, Volume *29*

Supplemental information

Fabricating a Cu-MBG-incorporated polyurethane foam with antibacterial properties and bioactivity for diabetic wound healing

Linqin Xie, Deng Huang, Kai Chen, Bangxuan Zhu, Yangkai Zhao, Jieke Li, Shouyi Wang, Mingliang Wu, and Yili Xiang

Supporting information

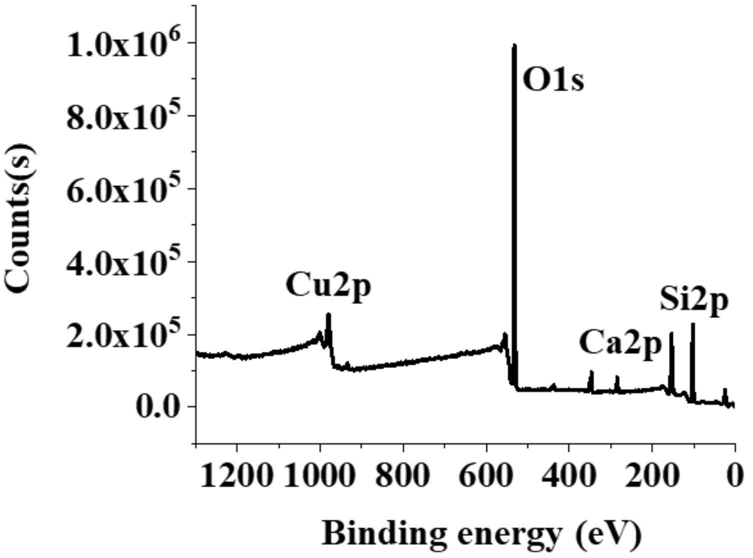

Fig. S1 XPS pattern of Cu-MBG.

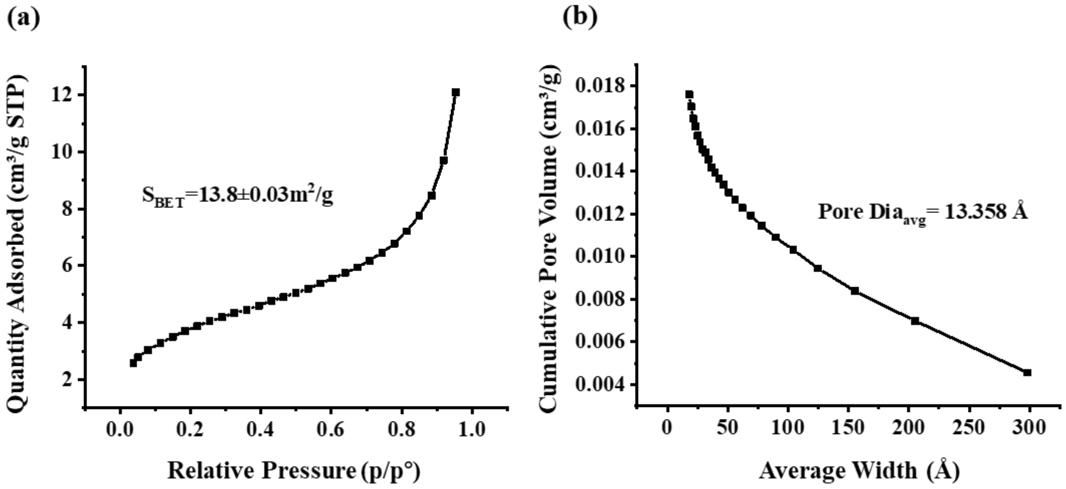

Fig. S2 (a) N_2 adsorption isotherms of Cu-MBGs. (b) Pore size distribution curves of Cu-MBGs.

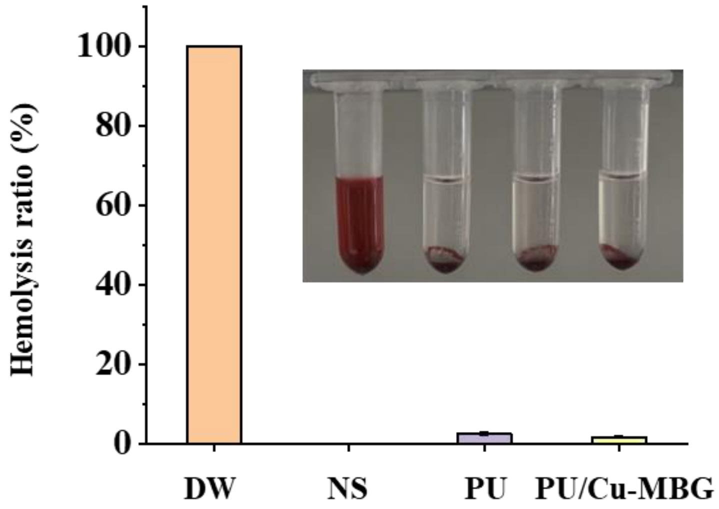

Fig. S3 Hemolysis ratio and representative hemolysis images for distilled water (DW), normal saline (NS), PU and PU/Cu-MBG.

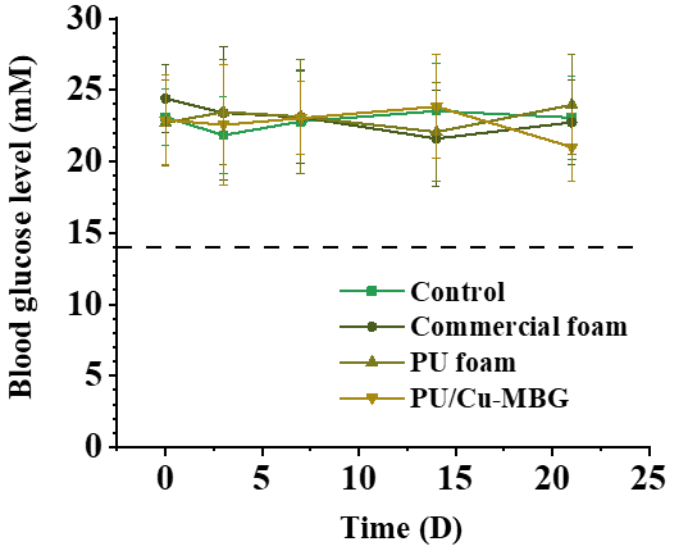

Fig. S4 Blood glucose level for each group during the experiment.

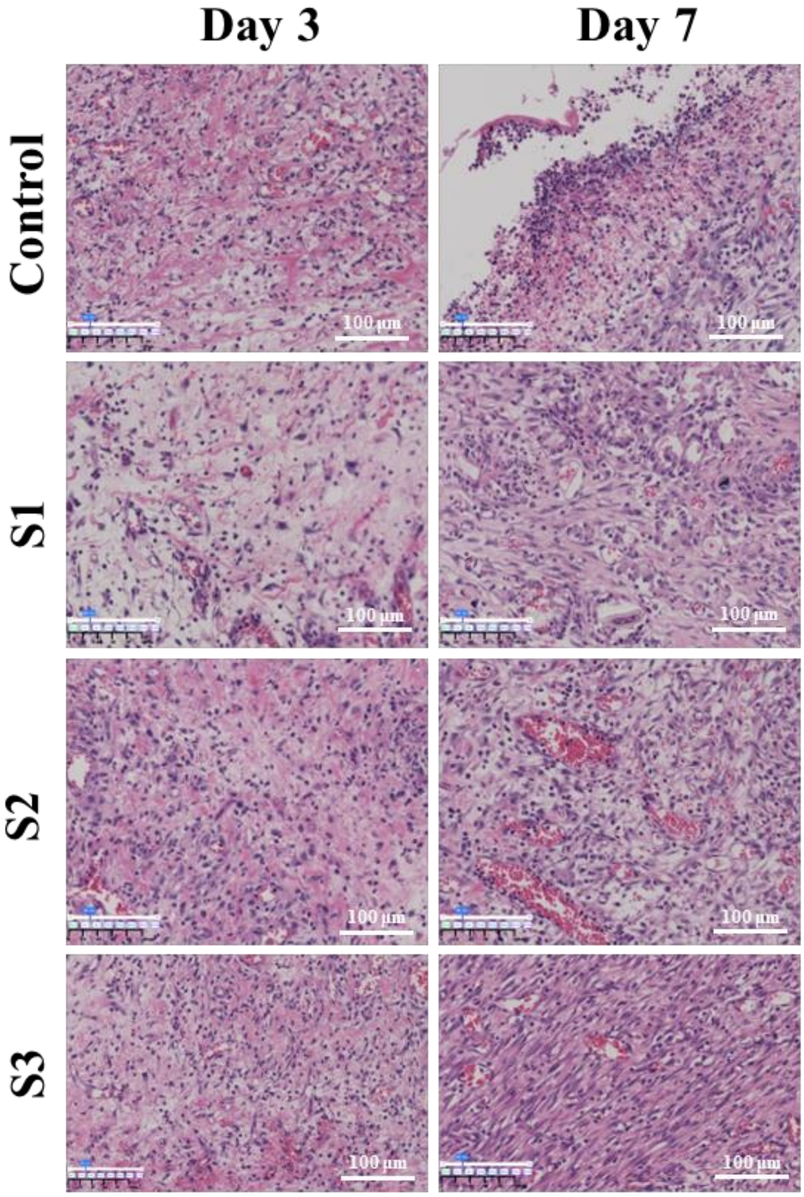

Fig. S5 At higher magnification, representative images of H&E staining of wound tissue sections from different treatment groups (S1: commercial foam, S2: PU foam, S3: PU/Cu-MBG) at days 3 and 7. Scale bar=100 µm.

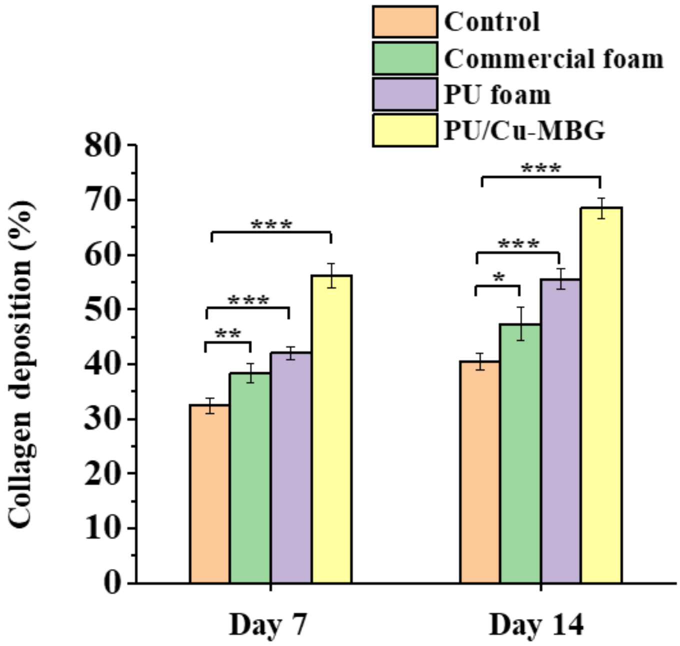

Fig. S6 Collagen deposition of different samples on days 7 and 14. Data are presented as mean ± SD (n = 5). (*p < 0.05, **p<0.01, ***p < 0.001.)

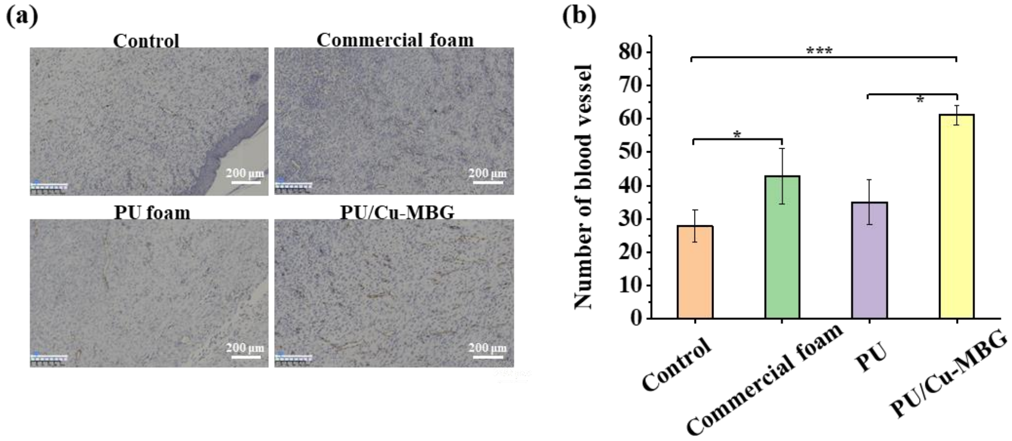

Fig. S7 (a) Representative immunohistochemical staining of CD31 in wound tissues on day 14. Scale bar=200 μm. (b) Quantitative analysis of CD31-positive micro-vessel density. Data are presented as mean ± SD (n = 5). (*p < 0.05, ***p < 0.001.)
